# Successional Development of Fungal Communities Associated with Decomposing Deadwood in a Natural Mixed Temperate Forest

**DOI:** 10.3390/jof7060412

**Published:** 2021-05-25

**Authors:** Clémentine Lepinay, Lucie Jiráska, Vojtěch Tláskal, Vendula Brabcová, Tomáš Vrška, Petr Baldrian

**Affiliations:** 1 Laboratory of Environmental Microbiology, Institute of Microbiology of the Czech Academy of Sciences, Vídeňská 1083, 14220 Praha 4, Czech Republic; lste906@aucklanduni.ac.nz (L.J.); tlaskal@biomed.cas.cz (V.T.); brabcova@biomed.cas.cz (V.B.); baldrian@biomed.cas.cz (P.B.); 2 The School of Biological Sciences, University of Auckland, Auckland 1010, New Zealand; 3 Department of Forest Ecology, The Silva Tarouca Research Institute for Landscape and Ornamental Gardening, Lidická 25/27, 60200 Brno, Czech Republic; tomas.vrska@vukoz.cz

**Keywords:** deadwood, decomposition, fungal community, succession, mixed natural forest, extracellular enzymes, fungal ecology

## Abstract

Deadwood represents an important carbon stock and contributes to climate change mitigation. Wood decomposition is mainly driven by fungal communities. Their composition is known to change during decomposition, but it is unclear how environmental factors such as wood chemistry affect these successional patterns through their effects on dominant fungal taxa. We analysed the deadwood of *Fagus sylvatica* and *Abies alba* across a deadwood succession series of >40 years in a natural fir-beech forest in the Czech Republic to describe the successional changes in fungal communities, fungal abundance and enzymatic activities and to link these changes to environmental variables. The fungal communities showed high levels of spatial variability and beta diversity. In young deadwood, fungal communities showed higher similarity among tree species, and fungi were generally less abundant, less diverse and less active than in older deadwood. pH and the carbon to nitrogen ratio (C/N) were the best predictors of the fungal community composition, and they affected the abundance of half of the dominant fungal taxa. The relative abundance of most of the dominant taxa tended to increase with increasing pH or C/N, possibly indicating that acidification and atmospheric N deposition may shift the community composition towards species that are currently less dominant.

## 1. Introduction

Fungi are the main contributors to deadwood decomposition [1,2]. In European forests where the volume of deadwood is on average 15.8 m^3^ ha^−1^ and can reach up to 880 m^3^ ha^−1^ [3], their role in ecosystem processes seems to be fundamental. By degrading deadwood, fungi contribute to stabilization of the wood carbon fraction by transforming it into soil organic matter [4,5,6], maintaining the fertility of forest soils [7] and promoting global biodiversity [8,9,10]. The communities of deadwood-associated fungi are often diverse [11,12] and include rare and endangered taxa [9].

Previous studies have shown that the composition of deadwood fungal communities could be affected by several environmental factors, namely, tree species [11], deadwood chemical properties [13] and deadwood size [14], as well as by the decay stage [15]. In certain surveys, tree species were previously indicated as the main drivers of deadwood fungal community composition [11,16,17], although this observation might not necessarily be valid in all instances [18].

Fungal communities on decomposing wood change during the process of decomposition [15,18,19,20]. Among the saprotrophic fungi mainly involved in deadwood decomposition, soft-rot ecological guilds contain pioneer species, while white-rot and brown-rot guilds appear to dominate during advanced decomposition [19,21,22]. Notably, the abundance of ectomycorrhizal fungi increases during later decay stages due to the increase in the contact surface between deadwood and soil which contains high amount of ectomycorrhizal fungi [18,23]. The evolution of total fungal biomass during wood decomposition shows various patterns, i.e., both an increase [18] and a decrease [24], depending on the substrate.

The abovementioned effects of tree species, deadwood chemistry and decay stage on decomposing fungi have usually been addressed at the community level. While some information is available on the successional preferences of certain fungal taxa [19,25], studies addressing the effects of other environmental factors are relatively rare. Moreover, the existing knowledge is largely based on fungal sporocarp surveys [11,26,27], an approach that has several important limitations: sporocarp production does not occur in all fungal species [24,28], and in others, it occurs only transiently at specific stages of the fungal life cycle [29] or under specific climatic conditions [27,30]. Such information is thus biased and not available for non-fruiting fungi.

This study used several analytical methods of both deadwood fungal communities and deadwood properties with the aim of describing the assembly of fungal communities during the decay process in a mixed natural forest and addressing questions about niche preferences of dominant fungal taxa. The composition of fungal communities in the deadwood of two tree species, *Fagus sylvatica* and *Abies alba*, was studied at four decay stages covering over 40 years of successive development. Fungal diversity was assessed by metabarcoding, with the nuclear ribosomal internal transcribed spacer 2 (ITS2) as a molecular marker. Fungal activity was approximated through quantification of extracellular enzymatic activities and assignment of putative ecology to fungal genera.

The specific objectives were (i) to characterize deadwood fungal communities by identifying their diversity, abundance and functional role over the decay process, (ii) to determine the relative importance of environmental drivers, i.e., decay stage, tree species and wood properties, to the fungal community composition, and (iii) to identify environmental drivers of the presence of dominant fungal taxa. We hypothesized that the composition of the fungal community differs between decay stages [18]. Tree species were expected to be the main driver of fungal community composition [11], and we expected wood chemistry to influence the fungal community composition in combination with decay stage [31]. The increase in nitrogen (N) content in deadwood during decomposition of *F. sylvatica* and *A. alba* [18] might be responsible for the distinction of early and late colonizers [25] and thus represent the most important chemical factor defining the niche space of individual fungal taxa.

## 2. Materials and Methods

### 2.1. Study Site

The study was carried out in the National Nature Reserve Salajka in the Outer Western Carpathians in the Czech Republic (49°24′ N, 18°25′ E). The reserve consists of a silver fir-beech forest of 21.9 ha. The mean annual temperature is 5.4 °C, and the mean annual precipitation is 1144 mm [32]. The elevation ranges between 715 and 820 m a.s.l. with an average slope of 17.3° [33]. No human disturbance, including the felling or removal of deadwood, has occurred since the protection of the area began in 1937 [34]. The living tree volume is 332 m^3^ ha^−1^ [35]. The dominant tree species is *Fagus sylvatica* (60.9% of the living tree volume), while *Abies alba* (29.2% of the living tree volume) and *Picea abies* (8.9% of the living tree volume) are also frequent [36]. Censuses of living and dead trees took place in 1974, 1994, 2007 and 2014, and allowed to build an exhaustive dataset of trees with a diameter at breast height (DBH) over 10 cm [37]. The mean amount of deadwood in the Salajka forest was estimated at 224 m^3^ ha^−1^ [35], of which *A. alba* represents 84.4% and *F. sylvatica* represents 11.9% [35,38].

### 2.2. Deadwood Selection and Sampling

The sampling of deadwood was performed on 14–16 October 2014. The selection of dead trees was made before the visit to the site to avoid subjectivity in the sampling. Two tree species, *F. sylvatica* and *A. alba*, and four decay classes were selected. Only trees with a DBH between 30 and 100 cm at the time when the trees were first recorded as fallen were considered. The decay classification was based on the year when the tree was first recorded as fallen. Trees that decayed while standing were excluded because the length of decomposition was unclear. The deadwood decay classes were designated as follows: <7 (recorded as fallen in 2014, <7 years of decomposition), 7–19 (recorded as fallen in 2007, 7–19 years), 20–40 (recorded as fallen in 1994, 20–40 years), and >40 (recorded as fallen in 1974, >40 years of decomposition). A total of 120 dead tree trunks (coarse woody debris, CWD) were randomly selected, ensuring even coverage of both tree species, and all decay classes were sampled (57 *F. sylvatica* CWD (<7, *n* = 15; 7–19, *n* = 18; 20–40, *n* = 16; and >40, *n* = 8) and 63 *A. alba* CWD (<7, *n* = 11; 7–19, *n* = 17; 20–40, *n* = 19; and >40, *n* = 16)).

For deadwood sampling, the length of each CWD (or the sum of the lengths of its fragments) was measured, and deadwood was collected at four positions (1/5, 2/5, 3/5 and 4/5 of the length) using a 40 cm drill with a 10 mm diameter bit. Before drilling, plants, lichens and surface bark were removed. The drill bit was sterilized with ethanol between drillings. Sawdust was collected in batches of two adjacent drill holes in sterile plastic bags and frozen at −20 °C within a few hours after drilling.

### 2.3. Chemical Properties of Deadwood

Sawdust material was weighed, freeze-dried and weighed again to estimate the dry mass content. Sawdust was then milled in an Ultra Centrifugal Mill ZM 200 (Retsch, Germany). After each sample, the parts of the centrifugal mill that were in contact with wood were properly washed with a cleanser and then ethanol before drying. The fine powder obtained after milling was used for the subsequent analyses.

The determination of pH was performed with an electronic pH meter model 3510 (Jenway, United Kingdom), according to an in-house protocol, i.e., after mixing wood powder in distilled water (1:10) and incubation for 24 h at room temperature. Carbon (C) and nitrogen (N) contents were measured at the Institute of Botany of the Czech Academy of Sciences (Průhonice, Czech Republic) as previously described in Větrovský and Baldrian [39]. C was measured using sulfochromic oxidation (ISO 14235), and N was estimated by sulfuric acid mineralisation with the addition of selenium and sodium sulfate and conversion to ammonium ions (ISO 11261), which were measured by a segmented flow analyser. The Klason lignin content was measured as the dry mass of solids after hydrolysis with 72% (*w/w*) H_2_SO_4_ [40].

### 2.4. Fungal Biomass and Extracellular Enzymatic Activities

The total ergosterol content, which approximates the fungal biomass, was extracted from 500 mg of freeze-dried wood powder using 10% KOH in methanol and analysed by high-performance liquid chromatography [41].

For the enzyme assays, 250 mg of freeze-dried wood powder was extracted with 12 mL of 50 mM acetate buffer (pH 5) for 2 h at 4 °C on an orbital shaker (100 rpm). The obtained extract was diluted with 6 mL of acetate buffer and used for the subsequent enzymatic analyses. The activities of β-glucosidase, β-xylosidase, β-galactosidase, cellobiohydrolase (exocellulase), α-glucosidase, *N*-acetylglucosaminidase, phosphomonoesterase (phosphatase) and esterase (lipase) were measured by a direct assay method as previously described [42]. Briefly, the sample extract was incubated with several substrates specific for each enzyme. These substrates were conjugated with fluorescent 4-methylumbellyferol, which allows the determination of enzyme activity based on the detected fluorescence. Fluorescence was recorded at an excitation wavelength of 355 nm and an emission wavelength of 460 nm. The activities of endo−1,4-β-glucanase (endocellulase) and endo-1,4-β-xylanase (endoxylanase) were measured with azo-dyed carbohydrate substrates (carboxymethyl cellulose and birchwood xylan, respectively) according to the substrate supplier’s instructions (Megazyme, Ireland). Briefly, 150 μL of dyed substrate was incubated with 150 μL of sample extract, and the amount of released dye was measured spectrophotometrically at 595 nm. The activities of laccase and manganese peroxidase were determined by evaluating the oxidation of 2,2-azinobis-3-ethylbenzothiazoline-6-sulfonic acid with a spectrophotometer at 420 nm and the oxidative coupling of 3-methyl-2-benzothizolinohydrazine and 3,3-dimetylaminobenzoic acid at 595 nm, respectively [42].

### 2.5. DNA Extraction and Sequencing Analysis of Fungal Community Composition

For DNA extraction, the two batches of sawdust initially collected for each CWD were kept separated. For each batch, total genomic DNA was extracted twice from 150 to 200 mg of freeze-dried wood powder with the NucleoSpin Soil Kit (Macherey-Nagel, Germany). The resulting four DNA extracts per log were pooled before PCR amplification. The ITS2 region of fungal rDNA was amplified in triplicate using the primers gITS7 and ITS4 [43], with pair-specific barcodes attached to both primers to exclude tag switching. The PCR mixture contained 1 μL of DNA at 5 ng μL^−1^ in a 25 µL final volume containing 2.5 μL of 10 × buffer for DyNAzyme DNA Polymerase, 1.5 μL of bovine serum albumin at 10 mg mL^−1^, 0.5 μL of 10 mM PCR Nucleotide Mix, 0.75 μL of DyNAzyme II DNA Polymerase at 2 U μL^−1^, 1 μL of 10 µM of each primer and 16.75 μL of H_2_O. The PCR amplification conditions were 94 °C for 5 min, 35 cycles of 94 °C for 30 s, 56 °C for 30 s and 72 °C for 30 s, followed by 7 min at 72 °C. The three PCR products were then pooled, purified using a MinElute PCR Purification Kit (Qiagen, Hilden, Germany) and quantified with a Qubit dsDNA High Sensitivity Assay Kit (Thermo Fisher Scientific, Waltham, MA, USA) on a Qubit 2.0 fluorometer (Life Technologies, Carlsbad, CA, USA). An amplicon library was prepared from the purified PCR products using a TruSeq DNA PCR-free Kit (Illumina, San Diego, CA, USA), and the resulting library was sequenced in-house on an Illumina MiSeq platform (2 × 250 base paired-end reads). Raw sequencing data have been deposited into the National Center for Biotechnology Information database under the accession number PRJNA693561.

### 2.6. Bioinformatic Processing of Sequencing Data

The sequencing data were processed using SEED 2 [44]. Only the forward reads were used for the analyses, as paired-end joining would exclude taxa with ITS2 region lengths higher than 400 bases, including abundant wood decomposing fungi from the genus *Armillaria* [45]. The sequences were filtered according to their quality, and sequences with a quality mean under 20 were excluded. The forward tags used for sample identification were removed. Then, sequences were de-replicated, the ITS2 region, even when incomplete, was extracted using ITSx 1.0.11 software [46], and sequences were re-replicated. Sequences shorter than 40 bases were removed, and chimeric sequences were identified using UCHIME [47] and removed. The remaining sequences were clustered in operational taxonomic units (OTUs) at a 97% similarity level using UPARSE [48]. Both algorithms were implemented in USEARCH 8.1.1861. For further analyses, the global singletons, i.e., the OTUs represented by only one sequence across the 120 samples, were ignored. The most abundant sequence was selected to represent each OTU and used for taxonomic identification. To identify the closest hits at the species level, the most abundant sequences were compared against the UNITE 8.1 database with BLASTn [49]. Non-fungal hits and unidentified sequences were deleted. The OTUs identified as the same species and having a similarity ≥ 97% with a coverage ≥ 95% were merged into single taxon, and species-level identification was used. For the OTUs with lower similarity, lower coverage or both, genus-level identification or the best available identification was used. The assignment of fungi to ecological categories was based on genus-level identification [50].

### 2.7. Statistical Analyses

Statistical analyses were performed using R v3.5.3 (R Development Core Team, R Foundation for Statistical Computing, Vienna, Austria). The results with *p* < 0.05 were considered statistically significant. All the values reported are means ± standard errors.

For the analyses of the diversity of fungal communities, the number of sequences for each CWD sample was randomly subsampled to 5000 sequences. The CWD samples having a sequencing depth below this threshold were not considered for the diversity analyses. The diversity indices were estimated with the ‘vegan’ R package [51].

The effect of tree species, decay class and their interaction on the chemical properties of deadwood, ergosterol content, enzymatic activities and diversity indices were tested by two-way ANOVA and Tukey-Kramer HSD tests or non-parametric Kruskal-Wallis tests. The effect of decay class was also evaluated for each tree species separately using one-way ANOVAs. Spearman’s correlations and general linear models were used to identify the relationships between ergosterol content and enzyme activities and ergosterol content and the carbon to nitrogen (C/N) ratio.

The composition of fungal communities was visualized with two-dimensional non-metric multidimensional scaling (NMDS) based on Bray-Curtis distances of relative abundances of OTUs using the ‘metaMDS’ function from the ‘vegan’ R package [51]. Two-way PERMANOVAs were used to test the effect of tree species, decay class and their interaction on fungal community composition, while one-way PERMANOVAs were used to test the effect of decay class for each tree species independently. The significance of environmental variables (decay class, pH, N, C, C/N, lignin and ergosterol) was analysed with the ‘envfit’ function of the ‘vegan’ R package [51], and the variables were fitted as vectors in the NMDS analyses.

Variation partitioning based on redundancy analyses was performed on Hellinger-transformed OTU abundances to identify the part of the variance explained by decay class, tree species or deadwood chemistry (pH, N, C, C/N and lignin) for the whole dataset and with decay class and deadwood chemistry for each tree species independently. The ‘varpart’ function from the ‘vegan’ R package was used [51]. The importance of the obtained variances was determined with Monte Carlo permutation tests.

The evolution of the similarity level of the fungal community composition was evaluated over the decay process for each tree species. For this, Mantel tests were used to examine the correlations between the OTU abundance matrix (Bray-Curtis dissimilarities of OTU relative abundances) and time matrix (Euclidean distance of time, in number of years, calculated from decay classes; [52]). The ‘mantel’ function of the ‘vegan’ R package was used [51].

For detailed analyses of the fungal taxa, the most abundant taxa were defined as those with >0.5% relative abundance in at least three CWDs or with >10% relative abundance in at least one CWD. Among the 208 most abundant taxa, 76 dominant taxa were selected for individual analyses. The 76 dominant taxa were those combining the two criteria chosen for the selection of the most abundant taxa. A species was considered associated with a certain tree species when it was recorded to have a relative abundance >0.1% in at least two CWDs. The effect of pH, C/N and their interaction on the abundance of these 76 taxa was evaluated with linear models.

## 3. Results

### 3.1. Deadwood Chemistry Over the Decomposition Process

**Figure 1:**
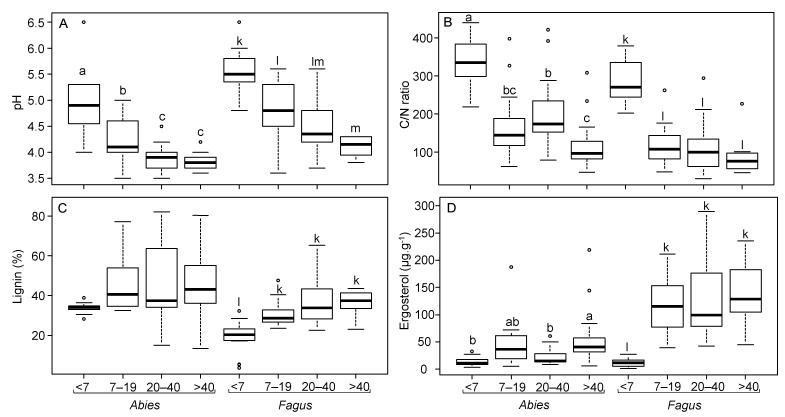
Chemistry of the coarse woody debris of *Abies alba* and *Fagus sylvatica* in the Salajka natural forest. (**A**) pH, (**B**) carbon to nitrogen (C/N) ratio, (**C**) percentage of lignin, and (**D**) ergosterol content. Different lowercase letters (designated a–m in the panels) indicate significant differences between decay classes according to Tukey-Kramer HSD tests performed for each tree species independently (*p* < 0.05). Gray Dots: outlier points.

The pH values of individual CWDs ranged between 3.5 and 6.5. Regardless of the tree species, pH decreased during deadwood decomposition (Figure 1A). The C/N ratio was highly variable, ranging from 30 to 439. For both tree species, the highest average C/N ratio was observed in the youngest CWD class: 338 ± 20 in *Abies* and 287 ± 15 in *Fagus*. For the older decay classes, the C/N ratios halved and did not significantly differ from each other (Figure 1B). The N contents were lowest in the youngest CWD, with 0.12 ± 0.01 mg g^−1^ and 0.15 ± 0.01 mg g^−1^ in *Abies* and *Fagus*, respectively. The lignin content in the *Abies* CWD varied considerably, from 13.3% to 82.2%, and was not significantly affected by decay length (Figure 1C). In *Fagus*, lignin content was significantly lower in the youngest CWD (Figure 1C).

### 3.2. Biomass and Functional Role of Deadwood Fungal Communities

Fungal biomass assessed through ergosterol content in the CWD was significantly affected by decay length in both tree species (Figure 1D). The youngest CWD had the lowest ergosterol contents (14.1 ± 2.7 µg g^−1^ in *Abies* and 11.9 ± 2.0 µg g^−1^ in *Fagus*). In *Abies*, the ergosterol content slowly increased in the older decay classes up to a maximal average of 57.6 ± 13.3 µg g^−1^ for CWD in the >40 class. In *Fagus*, the ergosterol content strongly increased in CWD in the 7–19 class, stabilizing at approximately 120 µg g^−1^ (Figure 1D).

Extracellular enzyme activity was differentially affected by decay length for both tree species (Figure S1). Out of the twelve enzymes assessed, only eight responded to environmental parameters, and the others, i.e., α-glucosidase, endocellulase, endoxylanase and laccase, were not affected by tree species, decay class or their interaction (0.15 < *p* < 0.98). β-glucosidase, β-galactosidase and cellobiohydrolase were affected by decay class for *Fagus* only, while β-xylosidase, *N*-acetylglucosaminidase, phosphomonoesterase and esterase were affected by decay length in both tree species (Figure S1). In *Fagus*, the lowest enzyme activities were measured for CWD in the <7 class, and the content then increased for CWD in the 7–19 class and tended to stabilize for CWD in the >40 class. In *Abies*, the enzyme content tended to be lowest for CWD in the <7 class and then increased for CWD in the 7–19 class before slightly decreasing for CWD in the 20–40 class; the exception was phosphomonoesterase, for which the content was highest in CWD in the 20–40 class. In *Abies*, the content of enzymes slightly increased in CWD in the >40 class. Manganese peroxidase was affected by tree species, with a higher activity in *Fagus* than in *Abies* (*p* < 0.001).

Spearman’s correlations showed significant positive correlations between ergosterol content and enzyme activities for all the enzymes except for endocellulase and α-glucosidase. The correlation between the C/N ratio and ergosterol was significantly negative (*p* < 0.0001, *R*^2^ = 0.39).

### 3.3. Composition of Deadwood Fungal Communities and Environmental Drivers

**Figure 2:**
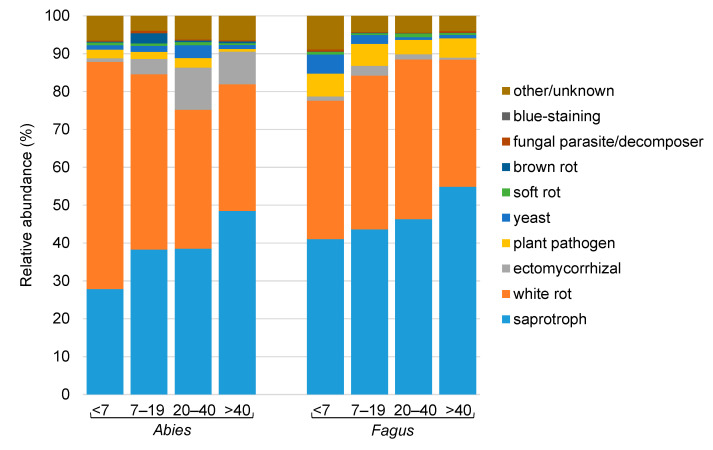
Relative abundances of ecological groups of fungi in the coarse woody debris of *Abies alba* and *Fagus sylvatica* from the Salajka natural forest.

The Chao−1 diversity index varied between 268 and 1279; in *Abies*, the fungal diversity in the youngest deadwood was significantly lower than that in the CWD older than 20 years, while in *Fagus*, the fungal diversity in the youngest deadwood was significantly lower than that in the CWD between 7 and 40 years old (Figure S2). Saprotrophic and white-rot fungi dominated the deadwood fungal communities, with average abundances of 42 and 41% of all sequences, respectively. Ectomycorrhizal fungi were significantly more abundant in *Abies* deadwood, while the opposite was true for plant pathogens (Figure 2).

**Figure 3:**
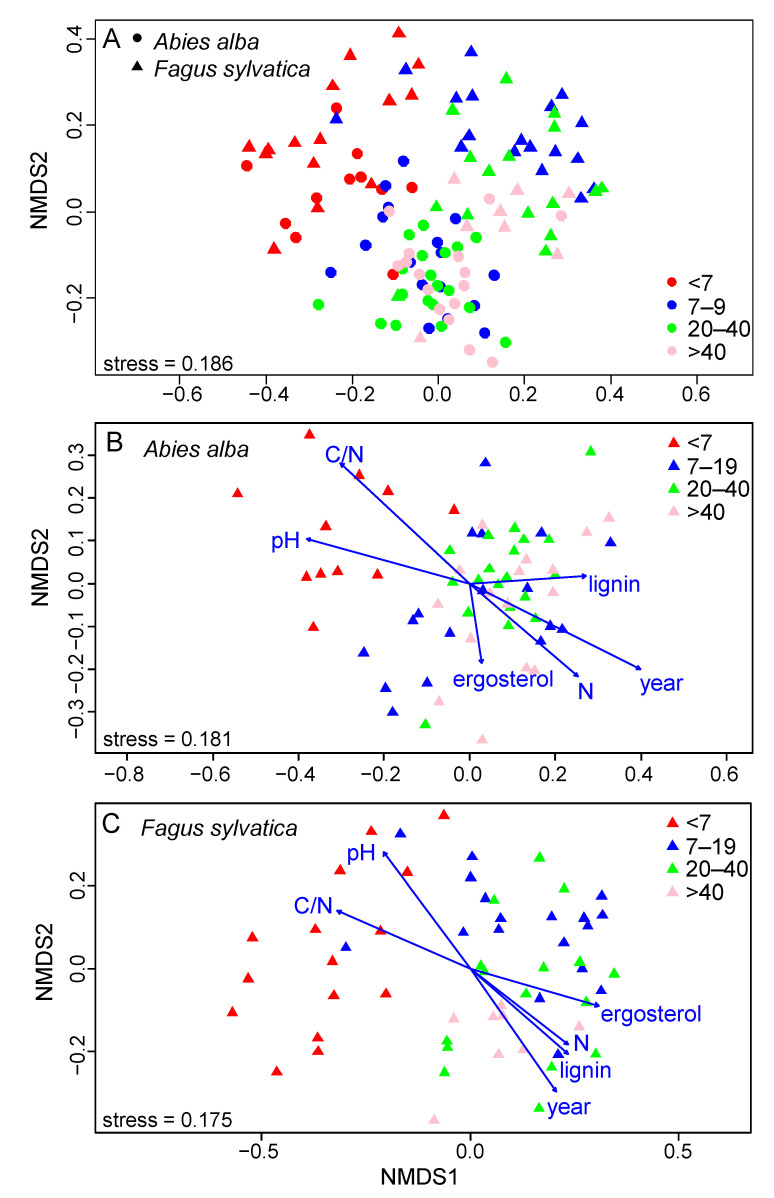
Two-dimensional NMDS of fungal communities in the coarse woody debris (CWD) of *Abies alba* and *Fagus sylvatica* from the Salajka natural forest. All CWD (**A**), CWD of *A. alba* (**B**) and CWD of *F. sylvatica* (**C**) with fitted environmental vectors. The vectors indicate the significant environmental variables.

The composition of the fungal communities in the deadwood was significantly affected by decay class (*p* = 0.001, *R*^2^ = 0.03), tree species (*p* = 0.001, *R*^2^ = 0.04) and their interaction (*p* = 0.001, *R*^2^ = 0.02). The fungal community composition tended to be similar for both tree species in the youngest decay class, while the fungal community composition was increasingly tree species-specific with increasing deadwood age (Figure 3A). For both tree species, similarities in the composition of the fungal communities decreased with increasing decay length (*p* < 0.001 for both tree species).

**Figure 4:**
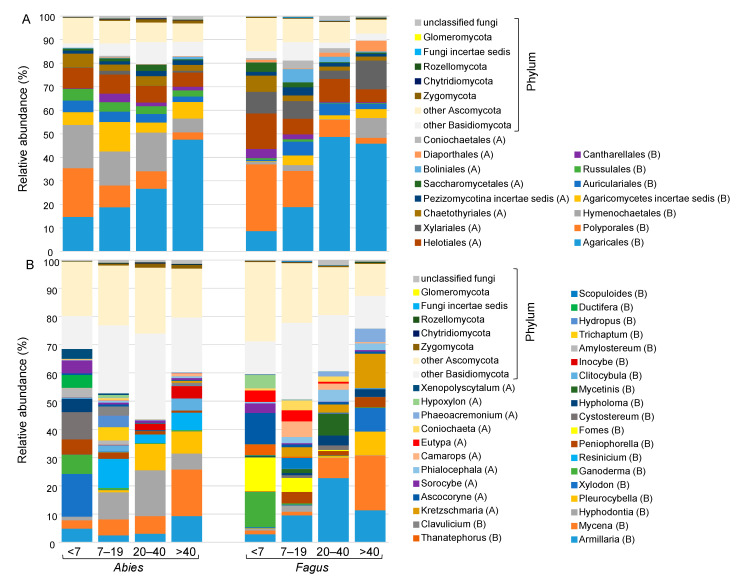
Relative abundances of fungal taxa in the coarse woody debris of *Abies alba* and *Fagus sylvatica* in the Salajka natural forest at the order level (**A**) and at the genus level (**B**). Abbreviations: A: Ascomycota; B: Basidiomycota. Only genera and orders with at least 3% relative abundance in one of the treatments were specified, and all others were classified to the phylum level.

The deadwood fungal communities were largely dominated by Basidiomycota (69.9%) and Ascomycota (28.3%), followed by Zygomycota (0.6%), Chytridiomycota (0.2%), Rozellomycota (0.1%) and Glomeromycota (<0.1%). Globally, the orders present in the older CWD were the same as those present in the young CWD in the <7 class, but the abundances changed with decay length. The abundance of the dominant order Agaricales increased with time, while that of the second most abundant order, Polyporales, decreased (Figure 4A). Some orders were more abundant for *Abies* than *Fagus*, e.g., Hymenochaetales, Agaricomycetes and Russulales, while some orders were more abundant for *Fagus* than *Abies*, e.g., Xylariales (Figure 4A). At the genus level, the composition of the fungal communities was most specific in the fresh deadwood (i.e., <7 years old), while that of the three older decay classes tended to be similar (Figure 4B, Figure S3). In addition, the dominant fungal genera were both tree- and decay stage-specific. *Ganoderma* was present in both tree species, with a high relative abundance in only the youngest deadwood. Several genera exhibited tree specificity, including *Eutypa* and *Fomes*, which were more abundant in *Fagus* CWD in the <7 and 7–19 classes, and *Resinicium*, *Inocybe* and *Hyphodontia*, which were abundant in *Abies* during certain stages of decomposition (Figure 4B, Figure S3).

The results of the variance partitioning showed that decay length, tree species and wood chemistry all had significant effects on fungal community composition. Most of the variation was explained by the joint effect of deadwood chemistry, i.e., pH, N, C, C/N and lignin, and tree species (2.4%), then by the pure effect of chemistry (2.2%) and by the joint effect of deadwood chemistry and decay class (1.9%). Only 1.1% and 0.6% of the variation was explained by the pure effects of tree species and decay class, respectively. A total of 91.7% of the variation remained unexplained by tested variables. Within both tree species individually, most of the variation in the fungal community composition was explained by the pure effect of deadwood chemistry, i.e., 3.7% for *Abies* and 4.8% for *Fagus*.

Finally, the significance of the environmental drivers fitted on the NMDS of each tree species (Figure 3B,C) demonstrated that the main drivers of the fungal community composition for both tree species were the decay length, followed by C/N and pH. In *Abies*, the N content, followed by the lignin content, was the significant driver of the fungal community composition, while in *Fagus*, the lignin content, followed by the N content, was the significant driver.

### 3.4. Dominant Fungal Taxa in Deadwood and Environmental Drivers

Out of the 9701 fungal taxa present in the whole dataset, 102 taxa exhibited sequence abundance higher than 10% in at least one CWD, and 106 additional taxa reached more than 0.5% in at least three CWD samples (Table S1). Among these 208 most abundant taxa, only 19 had relative abundances across the whole dataset higher than 1%.

Among the 76 dominant fungal taxa, i.e., those represented by more than 0.5% of relative abundance for at least three CWDs and accounting for more than 10% of all the sequences for at least one CWD (Table S2), 32 were white-rot fungi, 30 were saprotrophs, 3 were plant pathogens and another 3 were ectomycorrhizal fungi. Five fungal taxa—*Clavulicium macounii*, *Fomitopsis pinicola*, *Hydropus marginellus*, *Inocybe napipes* and *Phellinus hartigii*—were specific to *Abies*, and four—*Elmerina caryae*, *Hypholoma fasciculare*, *Kuehneromyces lignicola*, *Mycetinis alliaceus*—were specific to *Fagus*. Among the 26 fungal taxa that represented the most abundant species in more than two CWDs, 6 taxa were dominant in *Fagus*, 12 taxa were dominant in *Abies*, and the remaining 8 taxa were dominant in the CWDs of both tree species (Table S2).

**Figure 5:**
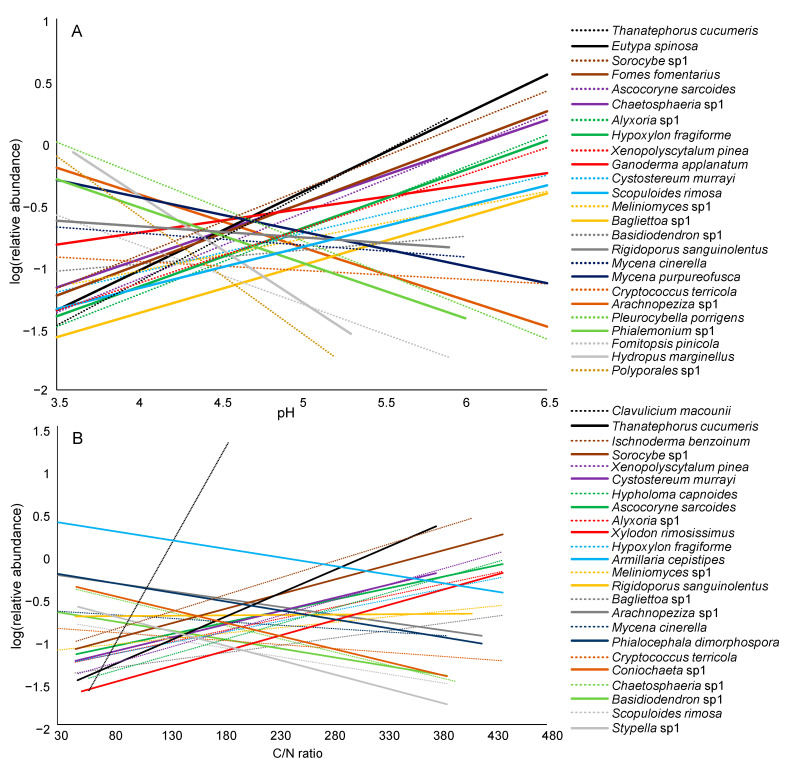
Linear fits of the relative abundances of dominant fungal taxa in the Salajka natural forest and environmental factors for those taxa significantly affected by pH (**A**) and by the C/N ratio (**B**).

**Figure 6:**
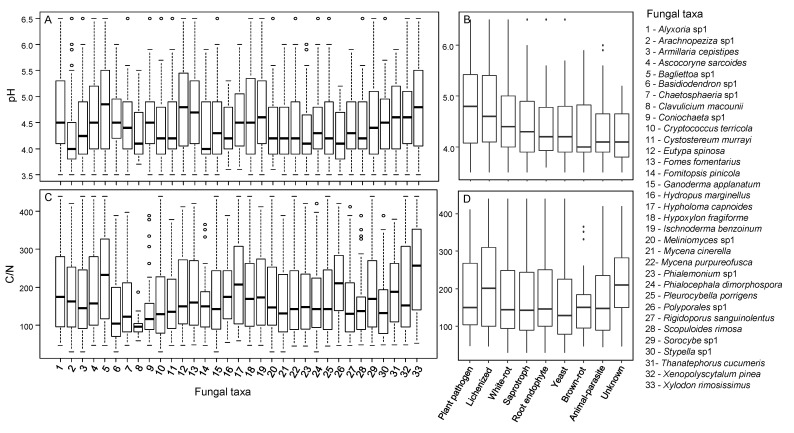
Values of pH (**A**,**B**) and C/N (**C**,**D**) for 33 dominant fungal taxa from the Salajka natural forest (left column) showing at least one significant effect among pH, C/N and their interaction. These taxa were classified at the ecological group level (right column).

The abundance of 33 of the 76 dominant fungal taxa responded significantly to pH, C/N or their interaction (Table S2). Twenty-five fungal taxa were affected by pH alone or its interaction with C/N, and 24 were affected by C/N alone or its interaction with pH (Table S2, Figure 5). However, the effects of pH and C/N on taxa abundance strongly differed between taxa (Figure 5). Out of 25 fungi responding to pH, the abundances of 15 taxa correlated positively with pH, while the abundances of 10 taxa showed a negative correlation (Figure 5A). Concerning C/N, an increase in abundance with the C/N ratio was recorded in 14 taxa, and a decrease was recorded in 10 taxa (Figure 5B). Plant pathogens and lichenized fungi showed the largest range of pH tolerance, while brown-rot fungi had the lowest range of C/N ratios (Figure 6).

## 4. Discussion

In this study, we characterized the deadwood fungal communities and wood chemistry over more than 40 years of wood decay. As previously observed for deadwood chemistry over the decomposition process [18,53], pH and the C/N ratio decreased over the decay length. The decrease in pH could partly result from the secretion of acidic components by wood-decomposing fungi [54,55,56] that acidify wood to increase the catalytic efficiency of their extracellular enzymes [57]. The decrease in the C/N ratio across deadwood decomposition reflected the relative increase in N content due to C loss through respiration [2,58]. Similar trends have also been demonstrated over deadwood decomposition periods for other tree species [59,60]. The C/N decrease may also reflect N fixation by bacteria [2,61] or N translocation from soil by fungal mycelia [61,62].

Fungal biomass content showed a negative correlation with the C/N ratio. Although fungi are able to colonize N-poor substrates, including deadwood, due to their low N requirements [1,63], the C/N ratio observed here in the youngest deadwood was very high, and its decrease definitely promoted fungal growth [64]. Ergosterol content is also positively correlated with the activity of all extracellular enzymes; this is not surprising if we consider that the vast majority of these enzymes are of fungal origin [2].

Although fungal biomass correlated with enzyme activity, no significant clear correlation was found between fungal diversity, assessed through the Chao-1 index, and fungal biomass and activity. Nevertheless, in the young CWD (i.e., in the <7 class), the diversity of the fungal community was low in both tree species, consistent with the lowest biomass and the lowest activity of all enzymes. This may be due to the low N content, which limits many fungal taxa. Some fungi, such as the fungal endophytes of living trees—mutualists, pathogens or neutral taxa—are, however, able to cope with this limitation [65] and often turn into saprotrophs after tree dieback [1,65,66]. Thus, both the fungal community diversity and their decomposition activity are enhanced in older decay stages. Globally, we observed an increase in diversity over the decay length, a pattern previously observed [15,28], yet not commonly valid [18].

Lignin showed a significant increase only in *Fagus*, with a slight increase in the CWD in the 7–19 class compared to the CWD in the <7 class. The relative content of lignin usually rises over the decomposition period [67,68]. However, lignin is a more recalcitrant compound and is less easily degraded than cellulose and hemicellulose [22,69]. By looking at the enzymes involved in lignin degradation, i.e., laccase and Mn-peroxidase [42], we observed no significant variation over decay length, except that Mn-peroxidase was more highly expressed in *Fagus* than in *Abies*. This difference between tree species could be linked to the increase in lignin content for *Fagus*. However, it seems that the fungal communities preferentially degraded cellulose and hemicellulose, with a difference between tree species. Indeed, β-glucosidase, cellobiohydrolase and esterase were highly secreted in *Fagus* CWD in the 7–19 class, showing that the fungal communities degraded both cellulose and hemicellulose. In *Abies*, however, the pattern of enzymatic secretion revealed fungal communities preferentially degrading less recalcitrant wood compounds, such as xylan and hemicellulose.

Interestingly, most of the enzymes that were expressed followed the same trends over the decay length. In *Fagus*, the set of enzymes simultaneously expressed was higher than that for *Abies*, suggesting more efficient decomposition. This finding is consistent with that of Přívětivý et al. [31], who highlighted a higher decay rate for *F. sylvatica* (mean residence time of 39 years) than for *A. alba* (58 years). The simultaneous expression of many enzymes also suggested that the fungal communities were able to degrade all wood components at the same time.

Among the 208 most abundant fungal taxa, the majority were saprotrophs and white-rot fungi. Although our approach did not allow us to determine which taxa produced which enzymes, their ecological placement suggests their direct involvement in the decomposition of deadwood [70]. Some other taxa were endophytes, plant pathogens and ectomycorrhizal fungi that have been observed in living wood and here used deadwood as a source of nutrients [65,66]. The composition of the fungal community at the genus level confirmed high diversity and highlighted the heterogeneity of the community. Indeed, comparisons of individual CWDs from the same decay length for each tree species separately indicated that some genera dominated certain CWDs but were absent from others. Such heterogeneity between CWDs of the same tree species and the same decay length is likely the result of several factors: CWD size heterogeneity [29], fine-scale heterogeneity of the surrounding environment [68,71,72], or the stochastic assembly process of fungal communities on fresh deadwood with a strong priority effect [73]. Although some genera were present at all decay stages, many others were constrained to certain stages of decay, confirming the successional pattern of the deadwood fungal communities [19,20,23].

Notably, the fungal community composition of both tree species tended to be similar in the young CWD and showed divergent development in later stages. Previous studies demonstrated that tree species were the main driver of the fungal community composition in deadwood [11,16]. Here, we identified wood chemistry, mainly pH and C/N, as the main drivers of fungal community composition. Thus, the similarities in pH and C/N in the young CWD that we observed could explain the similarities in the fungal communities during these early stages. According to our findings, the differences in fungal communities between both tree species affected rare fungal species because most of the dominant fungal taxa were common to both tree species. These rare species might be mainly specialists, i.e., tree species-specific, as demonstrated by Moor et al. [74]. Such hypotheses highlight the existence of many rare species in deadwood [75,76] and the importance of tree species diversity to promote the global diversity of fungi in forest ecosystems [77]. Thus, our findings suggested that rare fungal species are largely involved in community dissimilarities, as already mentioned in Thorn et al. [78].

After identifying the main environmental drivers, i.e., pH and C/N, our aim was to determine how they affected the dominant fungal taxa that played a major role in deadwood decomposition. Interestingly, by looking at the response of individual taxa, we noticed high disparity. The abundance of approximately half of the dominant taxa was significantly affected by pH, C/N or their interaction. Moreover, contrary to our expectation that pH would be the strongest predictor [16], C/N was found to be equally important. Depending on the taxa considered, the ranges of pH and C/N were different. These results may help to estimate the suitable ecological niches of the dominant fungi [79] and to predict the fate of these important fungal taxa with ongoing climate change. Indeed, among the changes expected at the global scale, both an increase in atmospheric N deposition [80,81] and acidification of forests [82,83] are expected; the latter is already observed in the Salajka forest [34]. From our findings, most of the dominant taxa will be positively affected by an increase in pH but negatively affected by an increase in N. Nevertheless, we demonstrated that many dominant taxa were not affected by pH or C/N, so we can assume that the important decomposition functions will be maintained. How the decay length of trees will be affected remains an open question.

Although it is interesting to be able to predict the responses of individual taxa to future global change, additional work is necessary to predict the functioning of the whole deadwood fungal community. It was evident that plant pathogenic species were among the fungi with larger ranges of pH tolerance than brown-rot fungi or endophytes, for example, while brown-rot fungal species showed the narrowest range of C/N ratios. Such a broad ecological niche for plant pathogens has recently been demonstrated concerning temperature and precipitation tolerance [84]. Our results indicate that tree pathogens could also adapt more easily than other ecological groups to changes in pH. We also demonstrated that in the Salajka forest, plant pathogens were more abundant in the deadwood of *Fagus* than in that of *Abies*. Consequently, the differential response of fungal ecogroups to climate change could condition the response of the tree community, i.e., favour certain tree species over others, and thus reduce the species diversity of the plant communities. Such differential responses according to the tree species considered have already been demonstrated in some European forests as a result of global changes, such as temperature, precipitation or air pollution [37,85,86].

Finally, considering CWD from the same tree species and similar decay length but from two forests approximately 300 km from each other [18], we found different dominant taxa. Indeed, Baldrian et al. [18] studied the fungal community in deadwood of *F. sylvatica* and *A. alba* in the Zofin natural forest, which has quite similar vegetation and climatic conditions as the Salajka forest [36]. Certain taxa, e.g., *Fomes fomentarius*, *Resinicium furfuraceum*, *Kretzschmaria deusta* and *Eutypa spinosa*, were dominant in both forests, but several of the most commonly appearing, quantitatively dominant fungi were limited to Salajka: *Mycena purpureofusca*, *M. romagnesiana*, *Camarops sp*. and *Mycetinis alliaceus*. If spatial distances <20 km do not seem to strongly affect fungal communities [12], we show that beta diversity over distances of several tens to hundreds of km is high. Local climatic or edaphic differences between regions may explain this result. Climate has been shown to be the main driver of the global distribution of fungi [84]. In our study, the regional effect could be partly due to the difference in precipitation between the Salajka and Zofin forests [87]. Błonska et al. [4] found that humidity can affect the amount of N and C in deadwood, which can affect the composition of the fungal community. In addition, Přívětivý et al. [33] also demonstrated distinct rates of deadwood decomposition between Salajka and Zofin due to moisture differences. These observations underline the importance of protecting natural forests at multiple locations for the preservation of rare or locally endemic fungi. It should be noted that preservation of deadwood in forests is equally essential for fungal protection since deadwood fungal communities show little overlap with other habitats, including litter and soil [88], and a reduction in deadwood content may cause the loss of rare fungal taxa.

## 5. Conclusions

The study of deadwood fungal community composition in the natural fir–beech Carpathian forest of Salajka across >40 years of decay showed that wood pH and wood C/N played a determinant role. A decrease in C/N promotes both fungal wood colonization and enzymatic activity, while pH and C/N are the main drivers of the fungal community composition. Among the vast diversity of fungi colonizing the CWD, most of the dominant fungi were common to both *F. sylvatica* and *A. alba*. The dissimilarities between communities in both tree species are mostly due to rare and specialist fungal species, highlighting the importance of tree species diversity to enhance fungal diversity in forest ecosystems. The abundance of approximately half of the dominant fungal taxa involved in deadwood decomposition was significantly affected by pH, C/N and/or their interaction. However, the responses of these taxa to pH and C/N were variable, suggesting a difference in the response of these taxa to future decreases in the pH of forest soils or increases in N deposition. In the context of current and future global changes affecting forests, additional studies on the responses of dominant fungi combined with the study of environmental conditions will be required to predict the changes that could occur at the level of deadwood decomposition.

## Data Availability

The data presented in this study are available within the article and the supplementary materials.

## Supplementary Materials

The following are available online at https://www.mdpi.com/article/10.3390/jof7060412/s1, Figure S1: Activity of extracellular enzymes in the coarse woody debris of *Abies alba* and *Fagus sylvatica* from the Salajka natural forest. Figure S2: Chao-1 diversity estimates of total fungal species richness in the coarse woody debris of *Abies alba* and *Fagus sylvatica* from the Salajka natural forest. Figure S3: Relative abundances of fungal genera in the coarse woody debris of *Abies alba* and *Fagus sylvatica* from the Salajka natural forest. Table S1: The most abundant fungal taxa found in the coarse woody debris of *Abies alba* and *Fagus sylvatica* from the Salajka natural forest. Table S2: Dominant fungal taxa found in the coarse woody debris of *Abies alba* and *Fagus sylvatica* from the Salajka natural forest.

## References

[1] Baldrian P. (2017). Forest microbiome: Diversity, complexity and dynamics. FEMS Microbiol. Rev..

[2] Tláskal V., Brabcová V., Větrovský T., Jomura M., López-Mondéjar R., Monteiro L.M.O., Saraiva J.P., Human Z.R., Cajthaml T., da Rocha U.N. (2021). Complementary Roles of Wood-Inhabiting Fungi and Bacteria Facilitate Deadwood Decomposition. mSystems.

[3] Puletti N., Canullo R., Mattioli W., Gawryś R., Corona P., Czerepko J. (2019). A dataset of forest volume deadwood estimates for Europe. Ann. For. Sci..

[4] Błońska E., Lasota J., Piaszczyk W. (2019). Carbon and nitrogen stock in deadwood biomass in natural temperate forest along a soil moisture gradient. Plant Biosyst..

[5] Harmon M.E., Fasth B.G., Yatskov M., Kastendick D., Rock J., Woodall C.W. (2020). Release of coarse woody detritus-related carbon: A synthesis across forest biomes. Carbon Balance Manag..

[6] Harris N.L., Gibbs D.A., Baccini A., Birdsey R.A., De Bruin S., Farina M., Fatoyinbo L., Hansen M.C., Herold M., Houghton R.A. (2021). Global maps of twenty-first century forest carbon fluxes. Nat. Clim. Chang..

[7] Sterkenburg E., Bahr A., Brandström Durling M., Clemmensen K.E., Lindahl B.D. (2015). Changes in fungal communities along a boreal forest soil fertility gradient. New Phytol..

[8] Vítková L., Bače R., Kjučukov P., Svoboda M. (2018). Deadwood management in Central European forests: Key considerations for practical implementation. For. Ecol. Manag..

[9] Nordén J., Abrego N., Boddy L., Bässler C., Dahlberg A., Halme P., Hällfors M., Maurice S., Menkis A., Miettinen O. (2020). Ten principles for conservation translocations of threatened wood-inhabiting fungi. Fungal Ecol..

[10] Baldrian P., Větrovský T., Lepinay C., Kohout P. (2021). High-throughput sequencing view on the magnitude of global fungal diversity. Fungal Divers..

[11] Krah F.S., Seibold S., Brandl R., Baldrian P., Müller J., Bässler C. (2018). Independent effects of host and environment on the diversity of wood—inhabiting fungi. J. Ecol..

[12] Müller J., Ulyshen M., Seibold S., Cadotte M., Chao A., Bässler C., Vogel S., Hagge J., Weiß I., Baldrian P. (2020). Primary determinants of communities in deadwood vary among taxa but are regionally consistent. Oikos.

[13] Kahl T., Arnstadt T., Baber K., Bässler C., Bauhus J., Borken W., Buscot F., Floren A., Heibl C., Hessenmöller D. (2017). Wood decay rates of 13 temperate tree species in relation to wood properties, enzyme activities and organismic diversities. For. Ecol. Manag..

[14] Atrena A., Banelytė G.G., Læssøe T., Riis-Hansen R., Bruun H.H., Rahbek C., Heilmann-Clausen J. (2020). Quality of substrate and forest structure determine macrofungal richness along a gradient of management intensity in beech forests. For. Ecol. Manag..

[15] Rajala T., Peltoniemi M., Pennanen T., Mäkipää R. (2012). Fungal community dynamics in relation to substrate quality of decaying Norway spruce (*Picea abies* [L.] Karst.) logs in boreal forests. FEMS Microbiol. Ecol..

[16] Purahong W., Wubet T., Lentendu G., Hoppe B., Jariyavidyanont K., Arnstadt T., Baber K., Otto P., Kellner H., Hofrichter M. (2018). Determinants of deadwood-inhabiting fungal communities in temperate forests: Molecular evidence from a large scale deadwood decomposition experiment. Front. Microbiol..

[17] Leonhardt S., Hoppe B., Stengel E., Noll L., Moll J., Bässler C., Dahl A., Buscot F., Hofrichter M., Kellner H. (2019). Molecular fungal community and its decomposition activity in sapwood and heartwood of 13 temperate European tree species. PLoS ONE.

[18] Baldrian P., Zrůstová P., Tláskal V., Davidová A., Merhautová V., Vrška T. (2016). Fungi associated with decomposing deadwood in a natural beech-dominated forest. Fungal Ecol..

[19] Rayner A.D.M., Boddy L. (1988). Fungal Decomposition of Wood: Its Biology and Ecology.

[20] Fukasawa Y. (2018). Fungal succession and decomposition of *Pinus densiflora* snags. Ecol. Res..

[21] Goodell B., Qian Y., Jellison J., Schultz T.P., Militz H., Freeman M.H., Goodell B., Nicholas D.D. (2008). Fungal decay of wood: Soft rot-brown rot-white rot. Development of Commercial Wood Preservatives.

[22] Bani A., Pioli S., Ventura M., Panzacchi P., Borruso L., Tognetti R., Tonon G., Brusetti L. (2018). The role of microbial community in the decomposition of leaf litter and deadwood. Appl. Soil Ecol..

[23] Rajala T., Peltoniemi M., Hantula J., Mäkipää R., Pennanen T. (2011). RNA reveals a succession of active fungi during the decay of Norway spruce logs. Fungal Ecol..

[24] Ovaskainen O., Schigel D., Ali-Kovero H., Auvinen P., Paulin L., Nordén B., Nordén J. (2013). Combining high-throughput sequencing with fruit body surveys reveals contrasting life-history strategies in fungi. ISME J..

[25] Lindahl B.D., Finlay R.D. (2006). Activities of chitinolytic enzymes during primary and secondary colonization of wood by basidiomycetous fungi. N. Phytol..

[26] Fukasawa Y., Osono T., Takeda H. (2009). Dynamics of physicochemical properties and occurrence of fungal fruit bodies during decomposition of coarse woody debris of *Fagus Crenata*. J. For. Res..

[27] Bässler C., Müller J., Dziock F., Brandl R. (2010). Effects of resource availability and climate on the diversity of wood-decaying fungi. J. Ecol..

[28] Kubartová A., Ottosson E., Dahlberg A., Stenlid J. (2012). Patterns of fungal communities among and within decaying logs, revealed by 454 sequencing. Mol. Ecol..

[29] Tomao A., Bonet J.A., Castaño C., de-Miguel S. (2020). How does forest management affect fungal diversity and community composition? Current knowledge and future perspectives for the conservation of forest fungi. For. Ecol. Manag..

[30] Kauserud H., Stige L.C., Vik J.O., Okland R.H., Hoiland K., Stenseth N.C. (2008). Mushroom fruiting and climate change. Proc. Natl. Acad. Sci. USA.

[31] Přívětivý T., Baldrian P., Šamonil P., Vrška T. (2017). Deadwood density and moisture variation in a natural temperate Spruce-Fir-Beech forest. Preprints.

[32] Tolasz R., Míková T., Valeriánová A., Voženílek V. (2007). Climate atlas of Czechia.

[33] Přívětivý T., Janík D., Unar P., Adam D., Král K., Vrška T. (2016). How do environmental conditions affect the deadwood decomposition of European beech (*Fagus sylvatica* L.)?. For. Ecol. Manag..

[34] Šamonil P., Vrška T. (2007). Trends and cyclical changes in natural fir-beech forests at the north-western edge of the Carpathians. Folia Geobot..

[35] Táborská M., Privetity T., Vrska T., Odór P. (2015). Bryophytes associated with two tree species and different stages of decay in a natural fir-beech mixed forest in the Czech Republic. Preslia.

[36] Král K., McMahon S.M., Janík D., Adam D., Vrška T. (2014). Patch mosaic of developmental stages in central European natural forests along vegetation gradient. For. Ecol. Manag..

[37] Vrška T., Adam D., Hort L., Kolář T., Janík D. (2009). European beech (*Fagus sylvatica* L.) and silver fir (*Abies alba* Mill.) rotation in the Carpathians—A developmental cycle or a linear trend induced by man?. For. Ecol. Manag..

[38] Král K., Valtera M., Janik D., Šamonil P., Vrška T. (2014). Spatial variability of general stand characteristics in central European beech-dominated natural stands–effects of scale. For. Ecol. Manag..

[39] Větrovský T., Baldrian P. (2015). An in-depth analysis of actinobacterial communities shows their high diversity in grassland soils along a gradient of mixed heavy metal contamination. Biol. Fertil. Soils.

[40] Kirk T.K., Obst J.R. (1988). Lignin determination. Methods Enzymol..

[41] Šnajdr J., Valášková V., Merhautová V., Herinková J., Cajthaml T., Baldrian P. (2008). Spatial variability of enzyme activities and microbial biomass in the upper layers of *Quercus petraea* forest soil. Soil Biol. Biochem..

[42] Baldrian P. (2009). Microbial enzyme-catalyzed processes in soils and their analysis. Plant Soil Environ..

[43] Ihrmark K., Bödeker I.T.M., Cruz-Martinez K., Friberg H., Kubartova A., Schenck J., Strid Y., Stendil J., Brandström-Durling M., Clemmensen K.E. (2012). New primers to amplify the fungal ITS2 region—evaluation by 454-sequencing of artificial and natural communities. FEMS Microbiol. Ecol..

[44] Větrovský T., Baldrian P., Morais D. (2018). SEED 2: A user-friendly platform for amplicon high-throughput sequencing data analyses. Bioinformatics.

[45] Větrovský T., Morais D., Kohout P., Lepinay C., Algora C., Awokunle Hollá S., Bahnmann B.D., Bílohnědá K., Brabcová V., D’Alò F. (2020). GlobalFungi, a global database of fungal occurrences from high-throughput-sequencing metabarcoding studies. Sci. Data.

[46] Bengtsson-Palme J., Ryberg M., Hartmann M., Branco S., Wang Z., Godhe A., De Wit P., Sánchez-García M., Ebersberger I., de Souza F. (2013). Improved software detection and extraction of ITS1 and ITS 2 from ribosomal ITS sequences of fungi and other eukaryotes for analysis of environmental sequencing data. Methods Ecol. Evol..

[47] Edgar R.C., Haas B.J., Clemente J.C., Quince C., Knight R. (2011). UCHIME improves sensitivity and speed of chimera detection. Bioinformatics.

[48] Edgar R.C. (2013). Uparse: Highly accurate OTU sequences from microbial amplicon reads. Nat. Methods.

[49] Nilsson R.H., Larsson K.H., Taylor A.F.S., Bengtsson-Palme J., Jeppesen T.S., Schigel D., Kennedy P., Picard K., Glöckner F.O., Tedersoo L. (2019). The UNITE database for molecular identification of fungi: Handling dark taxa and parallel taxonomic classifications. Nucleic Acids Res..

[50] Põlme S., Abarenkov K., Nilsson R.H., Lindahl B.D., Clemmensen K.E., Kauserud H., Nguyen N., Kjøller R., Bates S.T., Baldrian P. (2020). FungalTraits: A user-friendly traits database of fungi and fungus-like stramenopiles. Fungal Divers..

[51] Oksanen J., Blanchet F.G., Friendly M., Kindt R., Legendre P., McGlinn D., Minchin P.R., O’hara R.B., Simpson G.L., Solymos P. (2016). Vegan: Community Ecology Package. R Package Version 2.4-3. Vienna: R Foundation for Statistical Computing. https://CRAN.R-project.org/package=vegan.

[52] Franklin R.B., Mills A.L. (2009). Importance of spatially structured environmental heterogeneity in controlling microbial community composition at small spatial scales in an agricultural field. Soil Biol. Biochem..

[53] Arnstadt T., Hoppe B., Kahl T., Kellner H., Krüger D., Bauhus J., Hofrichter M. (2016). Dynamics of fungal community composition, decomposition and resulting deadwood properties in logs of *Fagus sylvatica*, *Picea abies* and *Pinus sylvestris*. For. Ecol. Manag..

[54] Hastrup A.C.S., Green F., Lebow P.K., Jensen B. (2012). Enzymatic oxalic acid regulation correlated with wood degradation in four brown-rot fungi. Int. Biodeter. Biodegr..

[55] Mäkelä M., Galkin S., Hatakka A., Lundell T. (2002). Production of organic acids and oxalate decarboxylase in lignin-degrading white rot fungi. Enzym. Microb. Tech..

[56] Takao S. (1965). Organic acid production by Basidiomycetes: I. screening of acid-producing strains. Appl. Microbiol..

[57] Baldrian P., Valášková V. (2008). Degradation of cellulose by basidiomycetous fungi. FEMS Microbiol. Rev..

[58] Rinne-Garmston K.T., Peltoniemi K., Chen J., Peltoniemi M., Fritze H., Mäkipää R. (2019). Carbon flux from decomposing wood and its dependency on temperature, wood N_2_ fixation rate, moisture and fungal composition in a Norway spruce forest. Glob. Chang. Biol..

[59] De Meo I., Lagomarsino A., Agnelli A.E., Paletto A. (2019). Direct and indirect assessment of carbon stock in deadwood: Comparison in Calabrian Pine (*Pinus brutia* Ten. subsp. *brutia*) forests in Italy. For. Sci..

[60] Lombardi F., Cherubini P., Tognetti R., Cocozza C., Lasserre B., Marchetti M. (2013). Investigating biochemical processes to assess deadwood decay of beech and silver fir in Mediterranean mountain forests. Ann. For. Sci..

[61] Rinne K.T., Rajala T., Peltoniemi K., Chen J., Smolander A., Mäkipää R. (2017). Accumulation rates and sources of external nitrogen in decaying wood in a Norway spruce dominated forest. Funct. Ecol..

[62] Philpott T.J., Prescott C.E., Chapman W.K., Grayston S.J. (2014). Nitrogen translocation and accumulation by a cord-forming fungus (*Hypholoma fasciculare*) into simulated woody debris. For. Ecol. Manag..

[63] Baldrian P. (2017). Microbial activity and the dynamics of ecosystem processes in forest soils. Curr. Opin. Microbiol..

[64] Di Lonardo D.P., van der Wal A., Harkes P., de Boer W. (2020). Effect of nitrogen on fungal growth efficiency. Plant. Biosyst..

[65] Song Z., Kennedy P.G., Liew F.J., Schilling J.S. (2017). Fungal endophytes as priority colonizers initiating wood decomposition. Funct. Ecol..

[66] Giordano L., Gonthier P., Varese G.C., Miserere L., Nicolotti G. (2009). Mycobiota inhabiting sapwood of healthy and declining Scots pine (*Pinus sylvestris* L.) trees in the Alps. Fungal Divers..

[67] Tláskal V., Zrůstová P., Vrška T., Baldrian P. (2017). Bacteria associated with decomposing dead wood in a natural temperate forest. FEMS Microbiol. Ecol..

[68] Kohout P., Sudová R., Brabcová V., Vosolsobě S., Baldrian P., Albrechtová J. (2021). Forest microhabitat affects succession of fungal communities on decomposing fine tree roots. Front. Microbiol..

[69] Martin A.R., Domke G.M., Doraisami M., Thomas S.C. (2021). Carbon fractions in the world’s dead wood. Nat. Commun..

[70] Noll L., Leonhardt S., Arnstadt T., Hoppe B., Poll C., Matzner E., Hofrichter M., Kellner H. (2016). Fungal biomass and extracellular enzyme activities in coarse woody debris of 13 tree species in the early phase of decomposition. For. Ecol. Manag..

[71] Král K., Janík D., Vrška T., Adam D., Hort L., Unar P., Šamonil P. (2010). Local variability of stand structural features in beech dominated natural forests of Central Europe: Implications for sampling. For. Ecol. Manag..

[72] Král K., Shue J., Vrška T., Gonzalez-Akre E.B., Parker G.G., McShea W.J., McMahon S.M. (2016). Fine-scale patch mosaic of developmental stages in Northeast American secondary temperate forests: The European perspective. Eur. J. For. Res..

[73] Hiscox J., Savoury M., Müller C.T., Lindahl B.D., Rogers H.J., Boddy L. (2015). Priority effects during fungal community establishment in beech wood. ISME J..

[74] Moor H., Nordén J., Penttilä R., Siitonen J., Snäll T. (2021). Long-term effects of colonization–extinction dynamics of generalist versus specialist wood-decaying fungi. J. Ecol..

[75] Dickie I.A., Wakelin A., Richardson S.J. (2020). Rare species of wood-inhabiting fungi are not local. Ecol. Appl..

[76] Kubartová A., Ottosson E., Stenlid J. (2015). Linking fungal communities to wood density loss after 12 years of log decay. FEMS Microbiol. Ecol..

[77] Purahong W., Wubet T., Krüger D., Buscot F. (2018). Application of next-generation sequencing technologies to conservation of wood-inhabiting fungi. Conserv. Biol..

[78] Thorn S., Chao A., Bernhardt-Römermann M., Chen Y.-H., Georgiev K.B., Heibl C., Müller J., Schäfer H., Bässler C. (2020). Rare species, functional groups, and evolutionary lineages drive successional trajectories in disturbed forests. Ecology.

[79] Davison J., Moora M., Semchenko M., Adenan S.B., Ahmed T., Akhmetzhanova A.A., Alatalo J.M., Al-Quraishy S., Andriyanova E., Anslan S. (2021). Temperature and pH define the realised niche space of arbuscular mycorrhizal fungi. New Phytol..

[80] Maaroufi N.I., De Long J.R. (2020). Global change impacts on forest soils: Linkage between soil biota and carbon-nitrogen-phosphorus stoichiometry. Front. For. Glob. Chang..

[81] Wang R., Goll D., Balkanski Y., Hauglustaine D., Boucher O., Ciais P., Janssens I., Penuelas J., Guenet B., Sardans J. (2017). Global forest carbon uptake due to nitrogen and phosphorus deposition from 1850 to 2100. Glob. Chang. Biol..

[82] Borůvka L., Mládková L., Penížek V., Drábek O., Vašát R. (2007). Forest soil acidification assessment using principal component analysis and geostatistics. Geoderma.

[83] Huang J., Mo J.M., Zhang W., Lu X.K. (2014). Research on acidification in forest soil driven by atmospheric nitrogen deposition. Acta Ecol. Sin..

[84] Větrovský T., Kohout P., Kopecký M., Machac A., Man M., Bahnmann B.D., Brabcová V., Choi J., Meszárošová L., Human Z.R. (2019). A meta-analysis of global fungal distribution reveals climate-driven patterns. Nat. Commun..

[85] Přívětivý T., Adam D., Vrška T. (2018). Decay dynamics of *Abies alba* and *Picea abies* deadwood in relation to environmental conditions. For. Ecol. Manag..

[86] Vašíčková I., Šamonil P., Fuentes Ubilla A.E., Král K., Daněk P., Adam D. (2016). The true response of *Fagus sylvatica* L. to disturbances: A basis for the empirical inference of release criteria for temperate forests. For. Ecol. Manag..

[87] Vašíčková I., Šamonil P., Král K., Fuentes Ubilla A.E., Daněk P., Adam D. (2019). Driving factors of the growth response of *Fagus sylvatica* L. to disturbances: A comprehensive study from Central-European old-growth forests. For. Ecol. Manag..

[88] Šamonil P., Daněk P., Baldrian P., Tláskal V., Tejnecký V., Drábek O. (2020). Convergence, divergence or chaos? Consequences of tree trunk decay for pedogenesis and the soil microbiome in a temperate natural forest. Geoderma.

