# Supplementary material for: Successional Development of Fungal Communities Associated with Decomposing Deadwood in a Natural Mixed Temperate Forest
Source: J Fungi (Basel). 2021 May 25;7(6):412. doi: 10.3390/jof7060412 (PMC8228407; doi:10.3390/jof7060412)
Supplement: Supplementary file 1 [file jof-07-00412-s001.zip › Supplementary Materials.pdf]

Supplementary Materials

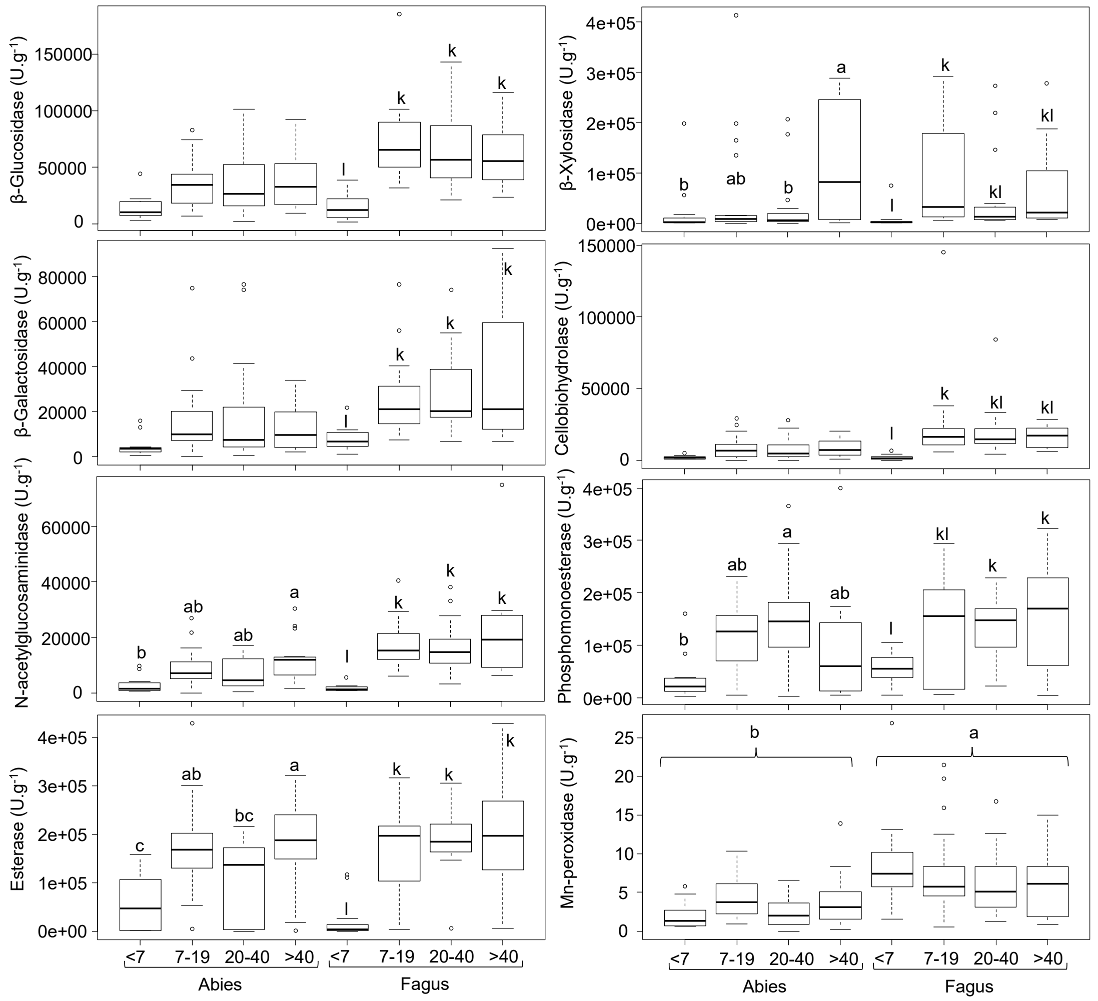

Figure S1. Activity of extracellular enzymes in the coarse woody debris of *Abies alba* and *Fagus sylvatica* from the Salajka natural forest. Different letters indicate significant differences between decay classes according to Tukey-Kramer HSD tests performed for each tree species independently ($P < 0.05$).

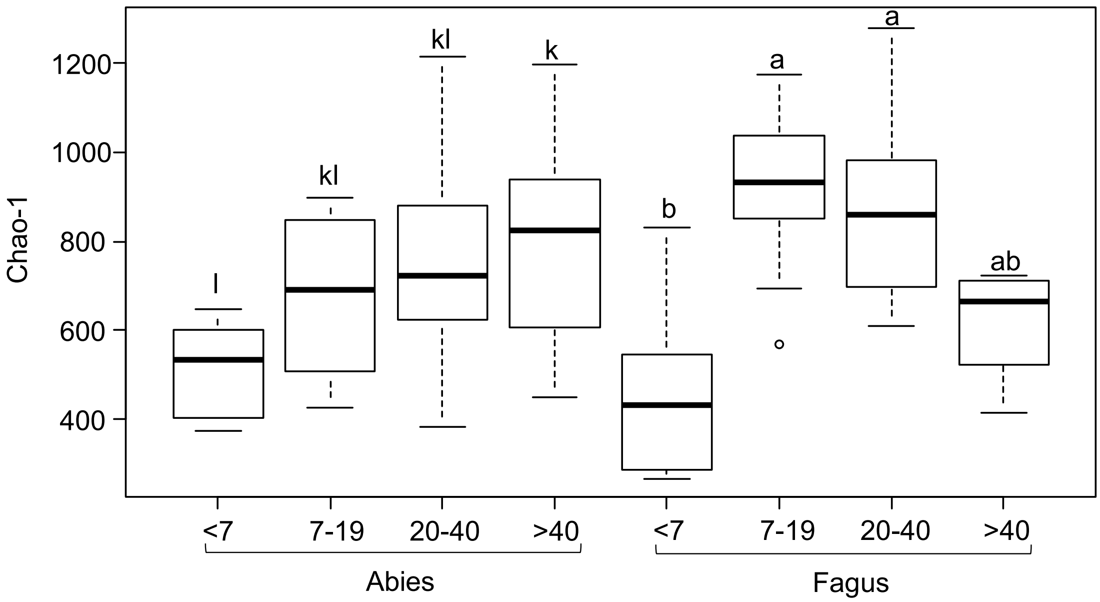

Figure S2. Chao-1 diversity estimates of total fungal species richness in the coarse woody debris of *Abies alba* and *Fagus sylvatica* from the Salajka natural forest. Different letters indicate significant differences between decay classes according to Tukey-Kramer HSD tests performed for each tree species independently ($P < 0.05$).

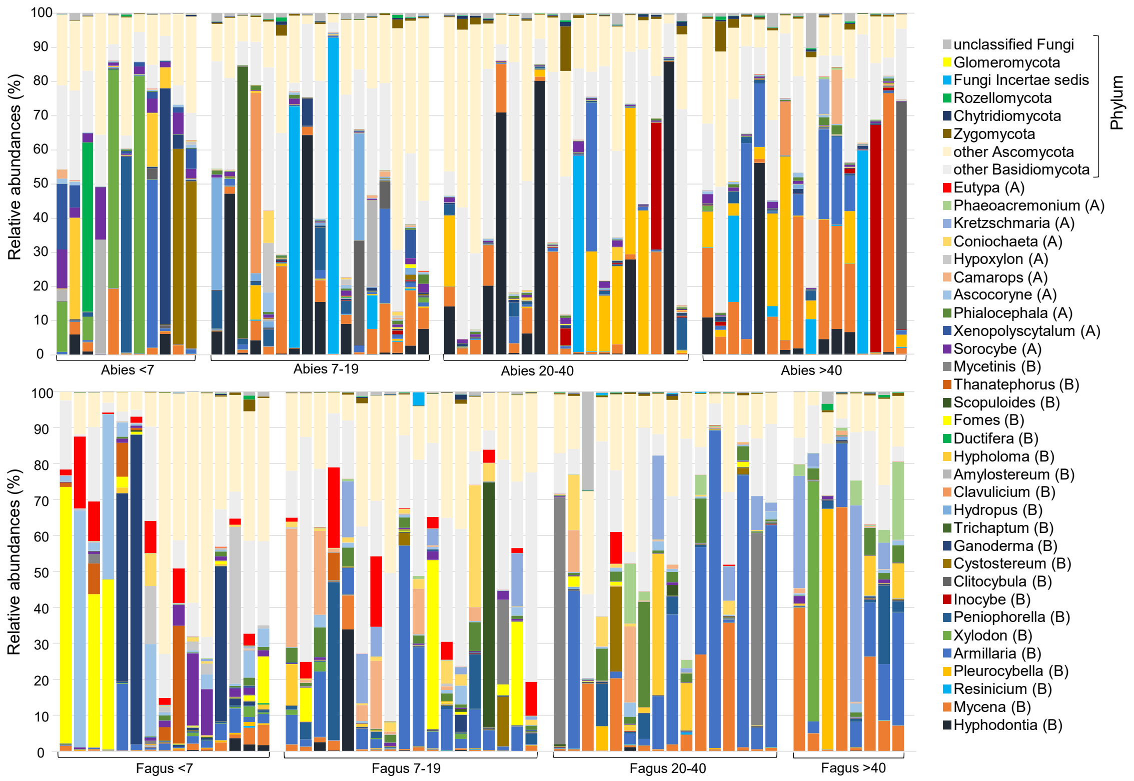

Figure S3. Relative abundances of fungal genera in the coarse woody debris of *Abies alba* and *Fagus sylvatica* from the Salajka natural forest. Abbreviations: A: Ascomycota; B: Basidiomycota. Only genera with at least 3% relative abundance in one of the treatments were specified, and all others were classified to the phylum level.

Table S1. The most abundant fungal taxa found in the coarse woody debris of *Abies alba* and *Fagus sylvatica* from the Salajka natural forest. The most abundant taxa correspond to taxa represented by more than 0.5% of relative abundance for at least three coarse woody debris samples or more than 10% of relative abundance for at least one coarse woody debris sample. The four decay classes represent the number of years since tree death.

→ See Excel file attached

Table S2. Dominant fungal taxa found in the coarse woody debris of *Abies alba* and *Fagus sylvatica* from the Salajka natural forest. Dominant taxa correspond to most abundant taxa represented by more than 10% of relative abundance for at least one coarse woody debris sample.

→ See Excel file attached
